# Editorial: Insights into the role of microorganisms on food quality and food safety

**DOI:** 10.3389/fmicb.2023.1237508

**Published:** 2023-07-13

**Authors:** Jinxuan Cao, Ying Wang, Changyu Zhou, Fang Geng

**Affiliations:** ^1^School of Food and Health, Beijing Technology and Business University, Beijing, China; ^2^College of Food and Pharmaceutical Sciences, Ningbo University, Ningbo, China; ^3^Meat Processing Key Laboratory of Sichuan Province, School of Food and Biological Engineering, Chengdu University, Chengdu, China

**Keywords:** microorganisms, food quality, food safety, volatile compounds, spoilage

With increasing health awareness, food safety and quality have been a growing demand globally. Food safety and quality depend upon many factors, including microbes in food production, processing, preservation, and storage. On the one hand, microbes such as bacteria, molds, and yeasts have a long history of application in food production, such as in the production of wine, beer, bread, and dairy products. On the other hand, the growth of microorganisms and contamination by microorganisms lead to food spoilage or even foodborne illness, threatening the development of the food industry.

Microorganisms play a crucial role in the production, preservation, and improvement of food. By transforming the chemical constituents of raw materials of plant/animal sources, functional microorganisms, particularly bacteria and yeast, can improve the sensory quality of food, enhance the bioactivity of nutrients, produce antioxidant and antimicrobial compounds, and promote food safety.

The physicochemical properties and the volatile flavor compounds of food are closely related to the nature of microorganisms, especially in fermented food. Liu, Cao et al. utilized a mixture of *yeast* and *Lactobacillus rhamnosus* YL-1 as a starter in the fermentation process of salami sausage, which could effectively decrease the degree of lipid oxidation and result in changes in flavor profiles. The combination of *Lactobacillus fermentum* YZU-06, *Staphylococcus saprophyticus* CGMCC 3475, and leucine has also been applied in the production of fermented sausage, which improves not only the diversity of flavor compounds but also the overall quality of sausages (Liu R. et al.). Liao et al. compared the quality and the main metabolic changes of instant dark teas fermented by different fungi, such as *Aspergillus cristatus, Aspergillus niger*, and *Aspergillus tubingensis*, revealing that the chemical constituents of instant dark teas were affected by the fungi. Tao et al. reviewed the utilization of microbiome in the fermentation process, which could remove the unpleasant beany flavors and enhance the aroma profile of plant-based meat analogs. Probiotics make a great contribution to gut health by improving digestion and enhancing nutrient absorption. Zhu Y. et al. demonstrated that feeding patterns can affect gut microbiota and the metabolites of Tibetan pigs, which further led to changes in meat quality.

The process of fermentation is influenced by several factors such as temperature, time, pH, oxygen levels, and microbial starter cultures. Food quality can be monitored through the regulation of fermentation conditions. Yu et al. found that the temperature of beef aging could affect the microbial community, physiochemical attributes, and flavor profiles of beef. Liu A. et al. observed significant differences between the bacterial community of vinegar from the same day with different fermentation depths, but no apparent difference appeared in the fungal community. In addition, the function of microbiota and volatile flavor compounds were affected by the microbial community at different depths. By comparing the physicochemical properties and microbial community compositions of Jinhua fat ham and lean ham, Zhang et al. explored the potential mechanism of characteristic microorganisms affecting the formation of flavor in lean dry-cured hams.

Spoilage and pathogenic microorganisms are considered one of the main factors threatening food quality and safety (Liu C. et al.; Guo et al.; Fulano et al.). Unscientific storage of food can cause an infestation of harmful microorganisms. For example, various fungal strains have been found in the production and preservation of dark tea, leading to the proliferation of fungi toxins. Xu et al. reviewed the contamination levels of common mycotoxin species, the main microbial sources of mycotoxin, and the possible ways to cause mycotoxin contamination in dark tea, providing a foundation for the prevention of harmful fungi.

To avoid illness and prevent food from spoiling, various technologies of food preservation have been developed to control the growth of microorganisms. Some microorganisms produce antimicrobial compounds and organic acids that inhibit the growth of spoilage-causing bacteria. Chen et al. found that the microbiota, mainly *Lactobacillus*, inhibited the formation of biogenic amines during the traditional fermentation of *Scomber japonicus*. To extend the shelf life of food, packaging materials have been explored to protect food from chemical and microbiological changes. Schmid et al. investigated the contaminating bacterial growth and survival in different fiber-based food packaging materials and evaluated the role of pH as an intrinsic antimicrobial factor. Furthermore, some active substances have been found to have inhibitory effects on the growth and toxicity production of microorganisms. Some *Aspergillus niger* produce ochratoxin A, which has harmful effects on human health, whereas tea polyphenols and epigallocatechin gallate were found to inhibit the growth of *Aspergillus niger* and ochratoxin synthesis, according to Zhao et al.'s study. Based on Propidium Monoazide combined with real-time PCR, Liu, Huang et al. developed a new method for fast detection of the antibacterial and bacteriostatic activity of disinfectants.

In conclusion, this Research Topic explored the beneficial and harmful effects of microorganisms on food quality and safety, highlighting the correlation between microbial community and volatile compounds. The reasonable application of beneficial microorganisms and the regulation of harmful microbial infestation are essential to achieve the desired properties, leading to reliable food products and ensuring food quality, safety, and consistency.

## Author contributions

YW, CZ, and FG collected literatures, organized information, and wrote the first draft of the article. JC provided writing ideas and checked and revised the first draft. All authors contributed to the article and approved the submitted version.

